# Some practical constraints and solutions for optical camera communication

**DOI:** 10.1098/rsta.2019.0191

**Published:** 2020-03-02

**Authors:** Weijie LIU, Zhengyuan Xu

**Affiliations:** CAS Key Laboratory of Wireless-Optical Communications, University of Science and Technology of China, Hefei, Anhui 230027, People’s Republic of China

**Keywords:** optical camera communication, covert communication, rolling shutter, region of interest, non-line of sight, optical wireless communication

## Abstract

Mobile wireless communication heavily relies on the radio frequency to convey message and data. However, its limited spectrum can hardly meet the demands for the future high data rate applications. Optical wireless communication, in particular visible light communication, opens up vast optical spectrum for communication, and meanwhile can retrofit the light sources as the communication transmitters in the existing working or living environments. In conjunction with the ubiquitous cameras in hand-held consumer electronics such as smartphones and pads, optical camera communication (OCC) further takes advantages of image sensors as the communication receivers and realizes low-cost communication systems. This article first provides an overview of OCC systems. It then addresses some practical constraints, ranging from sensor low frame rate and instability, rolling shutter readout, to visual qualities of displayed images and videos, and link blockage between the transmitter and receiver. Accordingly, it introduces existing and new solutions to deal with those constraints by data modulation, newly developed camera structures, post-processing of sensed signals and non-line of sight OCC as a new form. In particular, indirect paths by either the indoor surface reflection or the outdoor atmospheric scattering are explored for link connectivity under blockage. Finally, some future research directions are suggested.

This article is part of the theme issue ‘Optical wireless communication’.

## 1. Introduction

Traditional radio frequency (RF) communication techniques have been developed tremendously over the recent decades. Although it is not yet entirely clear what the next-generation technologies will provide, they aim at higher transmission speeds, lower latency, improved spectral efficiency, higher network capacity, and lower power consumption and cost [1]. Undoubtedly, the demands placed on the RF spectrum will continue to grow. To alleviate the RF spectrum shortage, higher frequency bands including millimetre wave [2], terahertz [3] and petahertz bands are all under consideration. Optical wireless communication (OWC) explores the infrared (IR) [4], ultraviolet (UV) [5] and visible light (VL) [6] frequencies for communications. Communication in both the visible and invisible light spectra can also be termed as petahertz communication for convenience.

In the VL sub-band, visible light communication (VLC) technologies were developed many years ago [7] and continued to be improved [8–10]. By using off-the-shelf light-emitting diodes (LEDs), they can simultaneously serve lighting and communication applications, including indoor high-speed internet access, information downloading, positioning, outdoor vehicular to infrastructure networking and underwater OWC for autonomous underwater vehicles (AUVs). Typically, the light beam diverges in space. A concentrator is usually employed in front of a photo-detector to collect more optical power and support a transmission rate up to several Gbps [11]. However, the path loss increases dramatically with distance, restricting long-range communication and outdoor vehicular communication. Meanwhile, beam directionality creates difficulties in communication on the move, and dense light sources have to be distinguishable by a mobile terminal. Different from VLC, light fidelity (LiFi) can adopt either IR, VL or UV in the backward path [12,13] while using VL in the forward path the same as VLC. Besides, LiFi can provide seamless terminal mobility and point-to-multipoint bidirectional communication services, which can be deployed easily within existing wireless networks. However, LiFi is vulnerable in outdoor scenarios and unable to support long-range communications. Therefore, free-space optical (FSO) communication emerges as a good candidate for achieving long transmission distance, ultra-high data rates and flexible coverage by beamforming techniques [14,15]. Leveraging the lower attenuation of optical signals in the IR spectrum while penetrating the atmosphere, FSO communication typically adopts an IR laser diode (LD) or other types of laser as the transmitter rather than an LED in VLC and LiFi, achieving several thousand kilometres via a point-to-point link [16]. However, it is sensitive to the link conditions, such as weather, atmospheric turbulence and physical obstructions.

The ubiquity of smart devices equipped with cameras in our daily life motivates study of optical camera communication (OCC) [17–21]. It employs image sensors assembled in pervasive consumer electronics, such as smartphones and iPads, as an alternative to photodiode (PD) or avalanche photodiode (APD)-based receivers. As a pragmatic version of VLC, it allows easier delivery of various services by the smart devices. Compared to other OWC technologies, OCC has the unique features such as the larger receiver field-of-view (FOV), spatial and wavelength separation ability, and low cost. In some applications such as indoor positioning and navigation, vehicle steering, and remote controlling, cost and accuracy may be the main factors to consider rather than data rate. Thus, OCC built upon existing cameras is an appealing solution. Besides, the IEEE standard work group 802.15.7 m is dedicated to revision of a formerly established IEEE VLC standard to incorporate new physical layers which support OCC functionalities and medium access control (MAC) modifications [22]. Comparisons of different OWC technologies are summarized in table 1, showing their key characteristics, respectively.

**Table 1.** Comparisons of different OWC technologies. OOK, on-off keying; IM, intensity modulation; PM, pulse modulation; CDMA, code-division multiple-access; OFDM, orthogonal frequency division modulation; CSK, colour shift keying; OAM, orbital angular momentum.

technologies	VLC	LiFi	FSO	OCC
standard	IEEE 802.15.7	IEEE 802.15.11 LC SG	well developed	IEEE 802.15.7 m
transmitter	LED/LD	LED/LD	laser	LED/screen
receiver	PD/APD/PIN/camera	PD	PD/APD	camera
spectrum	VL	IR/VL/UV	IR/VL/UV	IR/VL
modulation	OOK, IM, PM, OFDM, CSK, etc.	OOK, IM, OFDM, CSK, etc.	OOK, IM, PM, OFDM, OAM, etc.	OOK, IM, PM, OFDM, CSK, etc.
data rate	Mbps ∼ Gbps	kbps ∼ Gbps	∼Gbps	bps ∼ Mbps
distance	<100 m	<10 m	>1 km	<100 m
implementation complexity	moderate	moderate	high	low
computation complexity	low	low	moderate	high
path loss	moderate	moderate	high	low
robustness to interference	moderate	moderate	—	high
additional function	illumination, localization	illumination	—	imaging, localization
limitation	illumination constraints, limited range, vulnerable to mobility	limited use in outdoor, limited range	sensitive to weather and turbulence	low data rate

## 2. Overview of the optical camera communication technologies

**Figure 1.**
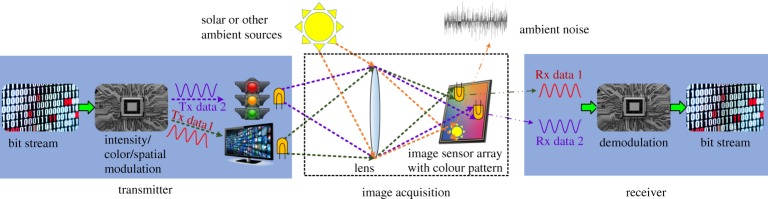
Operating principle of an OCC system. (Online version in colour.)

Figure 1 shows the basic principle of an OCC system. The bit stream can be modulated by intensity, colour or spatial position according to the requirement of an application scenario. The data streams transmitted from various light sources (e.g. a display screen or a digital traffic light) can be captured and distinguished simultaneously by using an image sensor. An image sensor typically comprises a two-dimensional array of PD pixels to convert the captured incident light into an array of electrical signals. Typically, the pixel outputs due to sunlight or ambient light sources contain only low-frequency constant components as compared to that generated by useful signal light sources. Thus, the desired signal corrupted by background noise sources (e.g. solar radiation or billboard) can be easily filtered out by a frame differential technique [23], in conjunction with narrowband optical filters.

**Figure 2.**
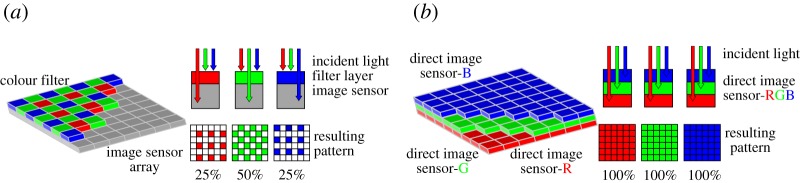
Colour filters of an image sensor. (*a*) Bayer arrangement. (*b*) Foveon X3 arrangement. (Online version in colour.)

To perform colour imaging, a Bayer pattern or Foveon X3 pattern-based colour filter array is generally placed above an image sensor array [10]. Figure 2*a* shows the Bayer pattern-based colour filter, containing 25%, 50% and 25% of red, green and blue filters, respectively. Figure 2*b* shows the Foveon X3 pattern-based colour filter, where the red, green and blue colours can be directly measured after penetrating corresponding colour filters. Such arrangement can avoid the loss of information transmitted by light. Moreover, since an image sensor can classify multiple light sources simultaneously due to its spatial resolution as illustrated in figure 1, it is a natural multi-element optical receiver with multi-colour channels for optical multiple-input multiple-output (MIMO) communication settings.

**Figure 3.**
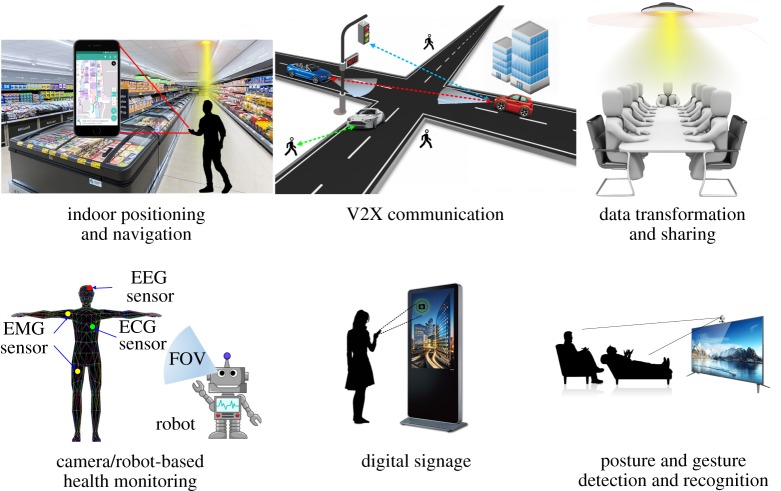
Some OCC application scenarios. (Online version in colour.)

The attractive features of image sensor receivers prompt tremendous research and application activities to realize new forms of sensing, communication and mobile computing. Figure 3 shows a few application scenarios of the OCC technologies. A commercial camera-based visible light positioning system can provide high positioning accuracy and is free of electromagnetic interference compared with a conventional RF-based positioning system. Vehicle-to-vehicle, vehicle-to-infrastructure and vehicle-to-pedestrian communications can be realized by OCC in an intelligent transportation system (ITS). Secure data sharing and transformation can be achieved by OCC as well. Health data collected by wearable sensors can be easily analysed by a robot equipped with cameras. Viewers can acquire more information by scanning the digital signage with their smartphones. Besides, posture and gesture are easily detected and recognized by OCC.

So far, there have been a few surveys on OCC in the literature. They concern about channel modelling, modulation and coding, and prospective business trends. In [17], the development, modulation scheme, superiority and shortcoming of the OCC systems were briefly reviewed. A new architecture was also proposed to handle the limitations including the camera sampling rate, frame rate variation and camera vibration. In [18], the authors provided a comprehensive overview of the OCC modulation schemes, and proposed an undersampled modulation scheme to achieve flicker-free for human eyes. In [19], the key technologies in IEEE 802.15.7r1, current research status, and appealing application scenarios of OCC were discussed in detail. The issues about designing and implementing a practical OCC system were addressed in [20]. Recently, Saeed *et al*. [21] provided a comprehensive survey on the major OWC techniques and their applications in navigation, positioning and motion capture.

Although OCC shows great potential in a range of applications, there still remain critical challenges limiting practical implementation. For example, most commercial cameras have a frame rate ranging from 30 fps to several hundred fps, which results in a data rate lower than several bps/pixel according to the Nyquist sampling theorem. Besides, transmitting at a low frequency by an LED source to match the low frame rate of the sensor is not acceptable for illumination and can be detected by human eyes. Moreover, the blocking of the optical link by buildings, and signal attenuation by rain and fog, will degrade the communication quality seriously.

In this work, we will first describe the aforementioned challenges and overview the corresponding solutions as comprehensively as possible. Some new application scenarios about both reflective and scattering non-line of sight (NLOS) OCC will be introduced. After that, we suggest some future research directions.

## 3. Frame rate constraints and solutions

As mentioned before, a commercial camera generally has a very low frame rate at about 30 fps. This limits the maximum symbol rate lower than 15 sps. Note that the critical flicker frequency (CFF) can be treated as the frequency at which a flicker light becomes indistinguishable from a constant light. The low frame rate will also cause a serious flicker effect considering the CFF for human eyes, which is typically 100 Hz [24]. Additionally, a commercial camera undergoes rate fluctuation due to sampling rate instability and asynchrony between the transmitter and the camera sampling clock. Therefore, in this section, we will summarize some key technologies to overcome the limitations of the constraints in either low frame rate or frame rate instability.

### (a) Data rate increase by modulations

**Figure 4.**
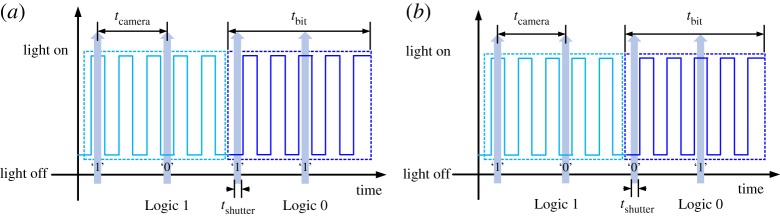
Undersampled intensity modulation. (*a*) UFSOOK signal with *f*_*s*1_/*f*_*s*0_ = 5:8. (*b*) UPSOOK signal. (Online version in colour.)

IEEE 802.15.7-2018 standardization confirmed some modulation schemes for low frame rate OCC from PHY layer IV to VI. Among various modulation schemes, Roberts from Intel firstly proposed undersampled frequency shift OOK (UFSOOK) [25–27], which uses two square wave patterns at two different high frequencies *f*_*s*1_ and *f*_*s*0_ much higher than the CFF to represent symbol ‘1’ and symbol ‘0’, respectively. Similarly, another undersampled scheme known as undersampled phase shift OOK (UPSOOK) was introduced by Luo *et al.* [28–30]. Two different bits are differentiated by the phase of the square wave. An example of UFSOOK and UPSOOK is shown in figure 4. The undersampled scheme is an innovative method for intensity modulation in OCC to avoid the flicker to human eyes, and can be used by off-the-shelf cameras with frame rate lower than 30 fps. The modulation can be easily generalized to high-order employing multiple frequencies or phases within a symbol period, generating a sequence of distinct states representing different symbols. Spatial two-phase shift keying (S2-PSK) can demodulate a randomly captured image by leveraging the spatial separation capability of the image sensor, providing a solution for a variable frame rate camera [31]. Twinkle-variable pulse position modulation (VPPM) and hybrid spatial phase shift keying (HS-PSK), specified in IEEE 802.15.7-2018 standard PHY IV, were proposed by Intel as well. In Twinkle-VPPM, bits are mapped by VPPM into one of two duty cycles, while the twinkle is produced by alternating between two duty cycles to generate a low-rate amplitude change for ROI signalling. In HS-PSK, a high rate data bit stream is modulated by dimmable spatial eight-phase shift keying (DS8-PSK) while the dimming level is controlled by S2-PSK. Several hybrid modulations standardized in PHY V and PHY VI include rolling shutter-FSK (RS-FSK) [32], compatible m-ary frequency shift keying series (CM-FSK) [33,34] and compatible OOK (C-OOK) [32], mirror pulse modulation (MPM), asynchronous quick link (A-QL) [35], variable transparent amplitude shape code (VTASC) [35], sequential scalable two-dimensional colour (SS2DC), invisible data embedding (IDE) and hidden asynchronous quick link (HA-QL) [35].

Apart from these standard modulation schemes, researchers have proposed other modulations to match the low frame rate camera. Table 2 lists experimental data rate results of various modulation and coding techniques. It can be seen that modulation and coding techniques start to shift from traditional intensity domain to the colour domain [44,62,63] and spatial domain [31,61,64–71] to achieve higher spectrum efficiency but at a cost of higher implementation complexity and more interference. To gain a better understanding of how these two domains operate in the OCC, we give a brief introduction to two special cases: optical spatial modulation (OSM) and colour shift keying (CSK).

**Table 2.** Comparisons of different OCC modulation schemes. WDM, wavelength division multiplexing; PWM, pulse width modulation; CIM, colour intensity modulation; UPAMSM, under-sampled pulse amplitude modulation with subcarrier modulation; UQAMSM, under-sampled quadrature amplitude modulation with subcarrier modulation; SM-ST, spatially-modulated space-time; L-STC, layered space-time code; S-TCF, spatial-temporal complementary frames; PTM, pixel translucency modulation; SVM, spatial visual modulation; SDMT, spatial discrete multitone.

year	data rates	distance (m)	modulation	complexity	reference
2016	1.28 kbps	0.05–0.2	OOK	low	[36]
2018	1–3.1 kbps	0.35	OOK	low	[37]
2015	0.3 kbps	0.5	OOK	low	[38]
2013	10, 15, 20 Mbps	0.5, 1.2	OOK	low	[23]
2017	9 kbps	0.1	PWM	moderate	[39]
2016	84 kbps	4	PWM	low	[40]
2015	0.3–1.5 kbps	0.5	PWM	low	[41]
2016	0.62–1.35 kbps	1.5–5.5	hybrid OOK-PWM	moderate	[42]
2016	95 kbps	1.2	CIM and PAM	high	[43]
2016	126.7 kbps	1.4	CIM	high	[44]
2018	57.6 kbps	0.65–1	CIM	high	[45]
2014	12.8 kbps	0.5	CIM	high	[46]
2016	317.3 kbps	0.2	CIM	high	[47]
2016	11.52 kbps	2	CIM	high	[48]
2015	5.2 kbps	0.3	CSK	moderate	[49]
2014	0.24 kbps	0.5	CSK	moderate	[50]
2016	2.88 kbbs	0.1	WDM	low	[51]
2015	0.12–0.96 Mbps	0.12–0.24	colour barcodes	high	[52]
2014	0.15 kbps	12	UPSOOK	low	[28]
2015	0.1 kbps	50	UPAMSM	moderate	[53]
2015	0.25 kbps	1.5	UPAMSM	moderate	[29]
2015	0.15 kbps	0.6	UPSOOK and WDM	high	[54]
2016	0.5 kbps	1.5	UQAMSM	high	[30]
2014	1 kbps	30	SM-ST	high	[55]
2015	1 kbps	40–210	L-STC	high	[56]
2016	240 kbps	0.6	S-TCF	high	[57]
2015	0.8–1.1 kbps	0.3–1.5	PTM	high	[58]
2017	16.67 kbps	0.7–1.5	SVM	high	[59]
2006	1.344 Mbps	2	SDMT	high	[60]
2010	12 Mbps	10	OFDM	high	[61]

#### (i) Optical spatial modulation

The spatial domain can be explored to further increase the data rate under the low frame rate constraint. The OSM [31,64–68], typically used in LiFi [13], can be adopted in OCC as well [72]. Since an image sensor is composed of a large pixel array and an optical lens, its outputs specifically in terms of light intensity or luminance values are arranged in a square matrix to form a digital electronic representation of the scene. Each optical path between a light source and a single pixel in an image sensor can be regarded as a VLC link using PD as the receiver. Each pixel can record data instantaneously or successively depending on shutter type of camera (global shutter or rolling shutter). Thus, multiple light sources can be used to further increase the capacity of the OCC channel, and the spatial position (also called spatial domain) of these light sources can be used to convey more information. Positions of different light sources on the image plane are distinguishable thanks to the optical lens, which contributes to the spatial separation ability of an image sensor. Therefore, the two-dimensional PD architecture facilitates the detection of light source position and the luminance. In OSM, the data bits are conveyed into spatial domain of the light source array, and an image sensor can fully demodulate bits within the captured image based on its spatial separation capability.

**Figure 5.**
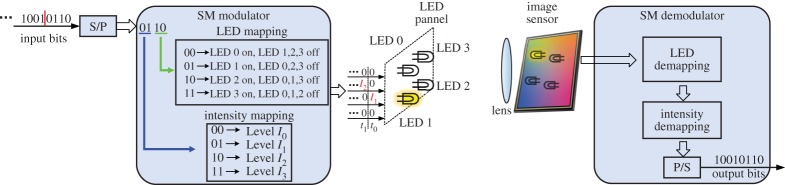
Demonstration of optical spatial modulation providing four bits per symbol. (Online version in colour.)

In an OSM-based OCC system using LEDs in the transmitter, a bit sequence is firstly transformed to a parallel form, then passed to a modulator. The modulator maps the parallel bits to the transmitting symbols according to the combination of input parallel bits. Some bits control selection of the LEDs, while all other bits control intensity levels of selected LEDs. Typically, only one LED is activated while other LEDs are set to be off at any particular time instant. Therefore, LED mapping and level mapping are performed jointly. Figure 5 depicts a typical OSM principle providing four bits per symbol based on four LEDs as a group. Four bits are grouped to construct one symbol. The first two bits are used to select one of the four LEDs and the second two bits are used to set one of the four intensity levels for that selected LED. For example, in the first symbol period *t*_0_, a random bits combination ‘0110’ means LED2 is selected to transmit according to the first two bits ‘10’ and this LED transmits a pulse with optical power at *I*_1_ based on the second two bits ‘01’ while other LEDs remain off. At the receiver, the image sensor performs two steps to demodulate transmitted bits. First, to demodulate the information from the transmitter light array, the coordinates of each light source have to be obtained by implementing a frame differential technique [23] or Hough circle detection method [44] on the label or training light sources at the head of a bit sequence. After that, the index and intensity of the activated light source in that time interval can be distinguished and measured. Only if both the index and the signal level are detected correctly, is the bit sequence decoded without errors. Similarly, one can easily find the activated LED and transmission level for the second symbol period corresponding to bit group ‘1001’ along the input bit sequence.

Obviously, OSM can entirely avoid inter-carrier interference (ICI) and provide a larger capacity while keeping a higher energy efficiency than conventional low-complexity methods for the MIMO systems. For an array consisting of *N* light sources with *L* luminance levels at the transmitter, and a camera with frame rate *f*_cam_ at the receiver, the aggregated data rate can be calculated as (log _2_*N* + log _2_*L*) × *f*_cam_ bps. However, the receiver requires perfect channel knowledge for data demodulation. This may impose a complexity constraint on the channel estimator. Besides, OSM offers only a logarithmical data rate increase with the number of transmit elements rather than linear. This might limit the spectral efficiency given a practical number of the light elements. Certainly, the changing frequency of the entire light source array cannot exceed the CFF to avoid flicker.

#### (ii) Colour shift keying

The VL spectrum contains rich colours. The RGB-type LEDs combine three primary colours: namely red, green and blue, and emit their corresponding light simultaneously. For OCC, colourful LEDs offer a new degree of freedom to represent more information bits. Together with the natural colour filter architecture in an image sensor for easy colour detection, CSK is an effective modulation scheme for cost-effective parallel OCC systems [49,50].

**Figure 6.**
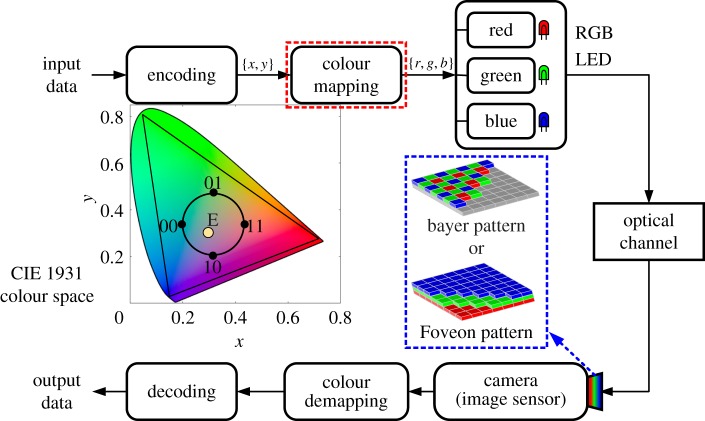
Block diagram of an OCC system using CSK modulation. (Online version in colour.)

As a unique modulation scheme specified in PHY III of the IEEE 802.15.7 standard, CSK is proposed to operate with RGB LEDs in order to enable higher-order spectrally efficient modulation and provide high data rate [73]. Unlike intensity modulation, CSK guarantees no intensity fluctuation of the luminary. According to the aforementioned two-dimensional PD array architecture and existing literature, CSK can be easily adopted in OCC without significantly increased complexity. Figure 6 depicts a typical OCC link using CSK modulation. Data are first mapped onto the constellation symbol $c_{i}=[x_{c_{i}},y_{c_{i}}]$, which is a member of the CIE 1931 *xy* chromaticity diagram. Then the symbol set is converted into the absolute luminous flux symbol set, $s_{i}=[r_{s_{i}},g_{s_{i}},b_{s_{i}}]$, in which $r_{s_{i}}$, $g_{s_{i}}$ and $b_{s_{i}}$ represent the absolute luminous flux of the red, green and blue colour bands, respectively. After that, the electric current levels required to generate the specific luminous flux of each symbol are determined to drive RGB LEDs, respectively. At the receiver, light passing through colour filters is recorded by the image sensor. Using the estimated channel matrix from pilot or training sequence, the receiver performs maximum-likelihood detection based on the received signal. Then the symbols are converted back into their *xy* chromaticity coordinates and decoded to bits eventually.

Clearly, CSK can entirely avoid intensity fluctuation of the luminary and improve the spectrum efficiency. For an RGB LED with *L* constellation points in the colour space, and a camera with frame rate *f*_cam_ at the receiver, the data rate can be calculated as *f*_cam_ × log _2_*L* bps. The cross-talk between colour channels is typically assumed to be deterministic and invertible. However, this may fail to be a valid assumption especially under sensor saturation and nonlinear distortion for the outdoor strong background radiation scenario, which imposes complexity constraints and channel estimation imperfectness on the receiver. Besides, the responsivity patterns of the pixels consisting of PDs typically vary with human perception, which may lead to a perceptible flicker for human eyes for a designed target intensity and colour. Furthermore, extra complexity will be imposed on the transceiver [74,75] to achieve a large dimension and high-quality constellation.

### (b) Data rate increase by exploring rolling shutter

An image sensor’s shutter determines how and when light gets recorded during an exposure. Typically, there are two methods to capture a static picture or each frame of a video signal. One is called the rolling shutter and the other called global shutter. Although both technologies record light within a necessary time interval, not every portion of the image starts and stops receiving light at the same time. As used in popular complementary metal oxide semiconductor (CMOS) cameras, a rolling shutter is exposed in a progressive step, in fact line by line from top to bottom.

**Figure 7.**
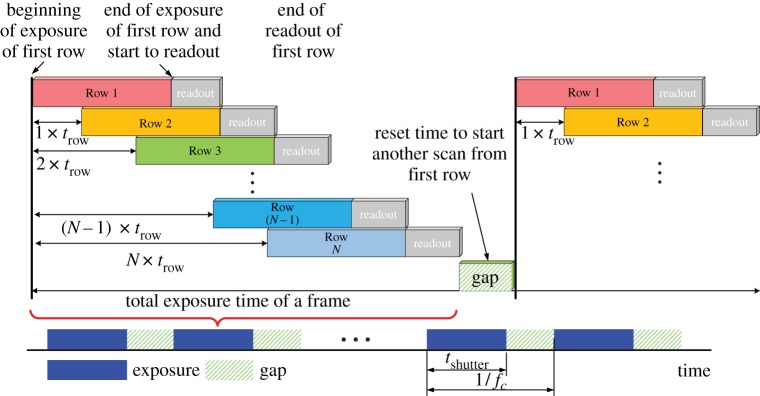
Frame capturing process of a rolling shutter camera. (Online version in colour.)

Figure 7 illustrates details of the rolling shutter process in successive image recording. As can be seen from this figure, the start and end of exposure on each row or column or individual pixel happen sequentially. So all the pixels are not exposed at the same time. It can take time up to 1/*f*_cam_ for all of the pixels on the sensor to finish exposure where *f*_cam_ is the frame rate. The effect will be noticed if the object is moving fast, causing a jello effect and damage to the image quality. However, one can take advantage of the rolling shutter mechanism in an OCC system to increase the sampling rate of the receiver [33,34,37,41,51,76–83].

**Figure 8.**
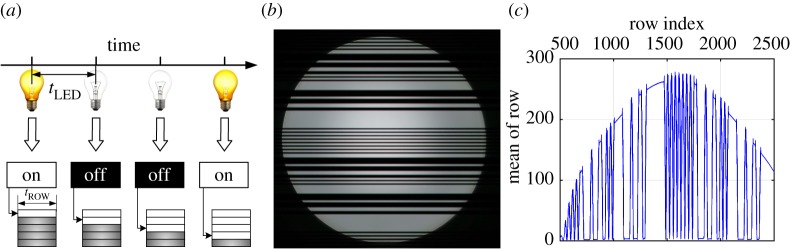
An operation process of rolling shutter. (*a*) Sequential data transmission by a commercial white LED at a rate of kHz. (*b*) A captured image (3968 × 2976). (*c*) The mean output of pixel values across rows of an image. (Online version in colour.)

Figure 8 demonstrates an operation process of an OCC system with sequential data transmission by the LED transmitter and signal reception by a rolling shutter-based sensor. Figure 8*a* shows sequential data transmission by a commercial white LED at a rate of kHz. When the LED is on, the first row of the image sensor starts to be exposed and read out before the exposure of the next row. Then the second row starts to be exposed as the LED is off. Such a process creates a bright stripe followed by a dark one. It continues till the last row. Finally, an image full of bright and dark stripes can be observed. The duration of LED’s states *t*_LED_ is typically set to be multiples of the line exposure time *t*_ROW_. Therefore, a stripe image is observed as shown in figure 8*b*, in which the bright and dark stripes can be easily separated. Figure 8*c* plots the mean output of the pixel values per row. Then the transmitted bits can be recovered by applying equalization and threshold detection.

Assume a camera (rows × cols = *M* × *N*) with frame rate *f*_cam_ at the receiver, taking *n* rows to represent the bit ‘1’ or bit ‘0’ to increase the signal-to-noise ratio (SNR). Then the data rate can be calculated as *f*_cam_ × (*M*/*n*/2) bps since there must be at least two complete slots in a frame to recover a correct data stream. Clearly, the rolling shutter mechanism improves the sampling rate greatly as compared to the traditional sampling method applied over the entire frame. The light source operates at a frequency of several kilohertz thanks to the high sampling rate, which makes it imperceptible to human eyes. Different techniques for improving the demodulation performance of the rolling shutter pattern were well addressed and investigated, such as blooming mitigation [79], extinction ratio enhancement [77] and optimal thresholding schemes [51]. Binary frequency shift keying as well as frequency division multiple access were proposed to enable multiple access while avoiding collision of signals from multiple LED transmitters [33].

However, due to the reset mechanism for preparing another exposure from last row to the first row, the gap within two successive frames showed in figure 7 may impose complex frame design and recovery especially while using the rolling shutter technique in receiving a long packet across multiple frames [79,84,85]. Besides, for a typical OCC operation setting, spatial distortion is inevitably visible in the rolling shutter mode especially while the image projected on the sensitive area is relatively small and under a mobile scenario. Despite the higher sampling rate, the link range of a rolling shutter-based OCC system is severely limited, and the bit rate changes with the distance and the size of the optical footprint [86,87].

### (c) Data rate increase by high-speed cameras

For intensity modulation with *N* levels in general, each symbol embeds log _2_*N* bits. If the camera frame rate is *f*_cam_, the bit rate becomes log _2_*N* × *f*_cam_/2 bps. It is clear that increasing the frame rate by directly using a high-speed camera can linearly increase the data rate. High-speed cameras can capture more information of fast-moving objects and changing objects in high frequency, compared to the normal cameras, opening a much expanded application space for commercial, industrial and military application sectors.

High-speed cameras at up to tens of thousands of *fps* can directly realize high-speed OCC and detection. For instance, Premachandra *et al.* [88] proposed an optical flow-based VLC tracking system by using an on-vehicle high-frame-rate (HFR) camera working at 1000 fps cooperated with an LED array changing at 500 Hz. Similarly, Ishii *et al*. [89] developed an HFR vision system operating in real time at 1000 fps for 1024 × 1024 pixel images. It implemented an improved optical flow detection algorithm on a high-speed vision platform. Such HFR vision system was designed for estimating optical flow rather than communication, but showed a great potential for OCC. To overcome the distance dependence of the LED to camera channel frequency response, Arai *et al.* [90] proposed a hierarchical coding scheme based on two-dimensional fast Haar wavelet transform for LED detection, and the communication system achieved a data rate of 128 kbps at distance 32 m with an HFR camera working at 4000 fps. Nagura *et al.* adopted a commercial camera with maximum frame rate 16 000 fps for LED array detection and communication, achieving a data rate of 128 kbps and 16 kbps for static mode and driving mode, respectively [91]. Nagura *et al.* [70] proposed two improved decoding methods to distinguish multi-valued data more reliably, leveraging a HFR camera (1000 fps) to achieve a data rate of 42.7 kbps at up to 65 m. Iwasaki *et al*. proposed a road-to-vehicle VLC system at intersection using an LED traffic light as the transmitter and an on-vehicle high-speed camera (1000 fps) as the receiver [92]. The system achieved the maximum data rate of 4 kbps with 64 LEDs modulated at 250 Hz.

**Figure 9.**
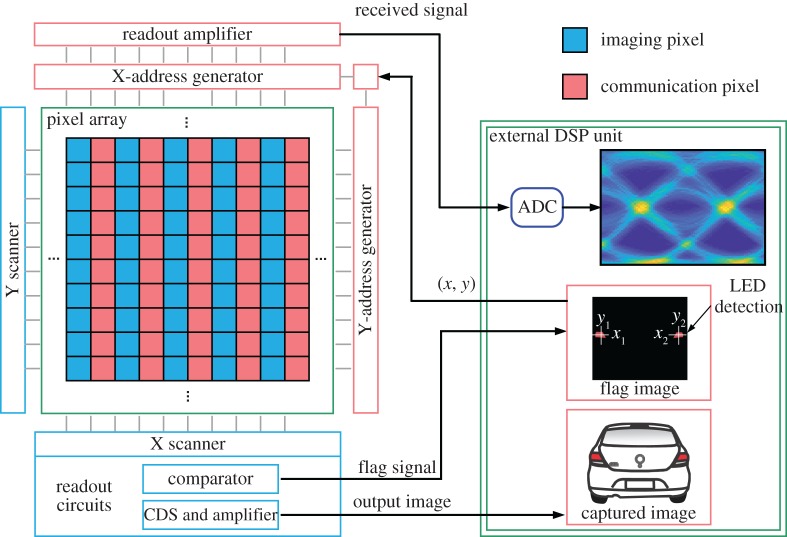
Structure of an OCI sensor and its operation process. (Online version in colour.)

To achieve high-speed data acquisition from various transmitters simultaneously, a new hybrid OCC and PD system, typically called optical communication image sensor (OCI), was proposed [23,69,93]. It is able to realize new types of image sensors with PD cells integrated between the imaging pixels. Figure 9 shows an OCI schematic and its operation process. An image output from image cells is used to detect and track various light sources. To avoid the intolerable computation cost of the image sensor output while the imaging pixels are running at a full resolution, a 1-bit flag for each imaging pixel is designed by using a comparator circuit. After an image processor acquires the flag image, the coordinates of the LEDs are determined by image processing techniques. Then the corresponding communication pixels are activated and the communication links are subsequently established according to the central coordinates. The output signal is amplified, digitized and demodulated in an external circuit. By using such novel architecture for the image sensor, the frequency response of the PD cells in the image sensor has been tested [69], and several PD communication methods have been implemented, including baseband modulations with line coding [23,93] and OFDM [69].

Currently, those high-speed cameras or hybrid OCI techniques are relatively expensive, but their demonstrated reliable performances of high-frame rate processing definitely pose them in unique positions towards future OCC applications.

### (d) Tackling the frame rate instability

**Figure 10.**
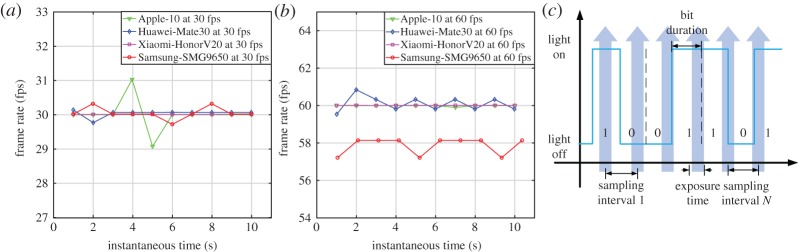
Measured frame rate variation concerning various types of smartphones operating at (*a*) 30 fps, (*b*) 60 fps and (*c*) its impact on the sampling process. (Online version in colour.)

Frame rate variation always exists in smartphone cameras. With the rapid development of consumer electronics, it almost becomes a standard configuration for a camera operating at 60 fps or higher at a 4K resolution, which facilitates the further improvement on the OCC techniques. However, due to the variation of sampling interval which relies on the manufacturing process and shutter mode of an image sensor, the synchronization turns out to be a necessary technique for an OCC system to recover bits from captured images. Figure 10 shows measured frame rate variation concerning various types of smartphones operating at 30 fps and 60 fps. It is clear that the practical frame rate results depart from the configured frame rates. Such mismatch causes sampling time errors and the receiver is out of synchronization as depicted in figure 10*c*.

To overcome the issue of unstable frame rate, Tian *et al.* simply adopted a four times oversampling by setting the camera frame rate at 330 fps, while the LED array remains at a 82.5 Hz refresh rate [43]. LightSync system was designed with a fast frame synchronization ability for a rolling shutter image sensor to minimize the synchronization error between an LCD screen and a camera [94]. Rajagopal *et al.* [33] proposed a preamble architecture and a periodic packet transmission scheme to guarantee the precise synchronization between the infrastructure and the camera operating at 30 fps. Hu *et al.* [49] investigated the impact of synchronization and proposed an error correction coding method to minimize the inter-frame packet loss. Different from other methods, Shiraki *et al.* [95] proposed a novel algorithm to demodulate the captured signal even without the information about transmitting period. Kwon *et al.* [96] proposed an effective synchronization strategy which maintains colour uniformity. They further investigated the colour independence of visual MIMO and improved the synchronization performance [97].

Existing synchronization strategies mainly depend on a reference aided signal or a reference code. However, the reference signal or reference code may cause a perceptible flicker or decrease the achievable data rate. Therefore, effective synchronization methods are necessary for further investigation and development in the OCC systems.

## 4. Visual constraints and solutions

There exist pervasive electronic displays such as smartphones and tablet screens, computer monitors, electronic advertising boards and TVs. Humans are more used to acquiring information from these electronic devices. With the ubiquity of these electronic display devices and cameras, a new OCC architecture called camera-screen communication (CSC) has emerged as a competitive solution for quick information acquisition.

A well-known CSC example, possibly as its predecessor, is the quick response (QR) code [98] which embeds information into two-dimensional barcodes. QR codes have become common in consumer advertising. In the past few years, there emerged various new QR-code-like tag forms with improved communication capabilities [52,61,99], enhanced reliability and security [47,94,100,101]. To explore more degrees of freedom, a COBRA system embedding five colours to achieve a higher capacity was designed for a colour barcode [99]. However, the usage of additional colours may lead to more decoding errors because of the colour cross-talks in the image sensor. In [52], an enhanced RainBar visual communication system was proposed based on a colour barcode system with a high-capacity barcode layout design, flexible frame synchronization and accurate code extraction. By means of multiple tracking bars and per-line tracking within each frame in conjunction with linear erasure coding, the LightSync system can recover lost frames and correct partial or mixed frames across the original frames.

However, these CSC systems mentioned above are perceptible for human eyes. From the perspective of visual quality, humans prefer to enjoy a normal full-screen viewing experience, while acquiring extra information from the screen to camera channel simultaneously. This kind of CSC is usually called covert CSC or hidden CSC, which is subject to a visual constraint for human eyes [32,58,59,102–108]. Unlike the traditional VLC system providing proper illumination while transmitting data simultaneously, a covert CSC system focuses more on the transmission while providing the quality display rather than illumination. Such a covert OCC protects data from any unintended attention based on an eye channel perceiving source contents and a sensor channel recovering transmitted data.

**Figure 11.**
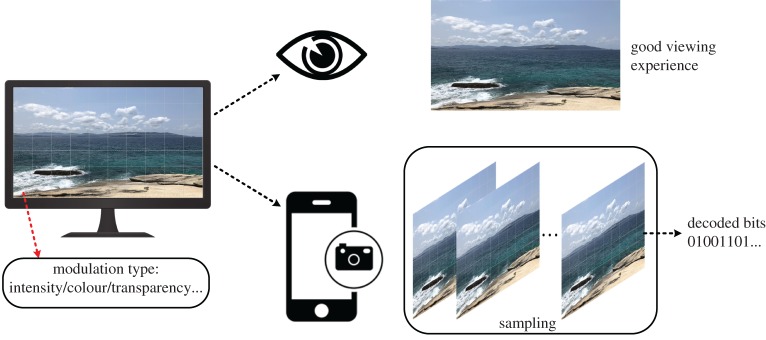
The concept of covert camera-screen communication. (Online version in colour.)

A general description of covert CSC is illustrated in figure 11. When data are encoded by intensity, colour or transparency as a single image or a stream of pictures displayed on a screen, a device equipped with a camera can capture the frame and then recover the transmitted data while guaranteeing an acceptable viewing experience. Such mutually conflicting requirements need to be fulfilled via optimizing communication performance and minimizing image distortions for human perception. Tremendous efforts have been made on this regard, aware of visual contents and communication performance.

Hilight [104] was first proposed by Li *et al.*, encoding bits into the orthogonal transparency channel on a smartphone screen and recovering bits by using another smartphone camera at a short distance (15 cm). Similarly, they realized a real time covert CSC system for any screen content, and discussed the impact of transmission distance, ambient light, hand motion and viewing angle [58]. Yuan *et al*. [103] proposed a novel algorithm to extract photographic messages from received images with photometric and geometric distortions in a visual MIMO optical system. Wang *et al.* [102] proposed an Inframe++ system which can support up to 360 kpbs by leveraging the spatial–temporal flicker–fusion property of human perception. Nguyen *et al.* [32] adopted content-adaptive encoding techniques to achieve a high-throughput and unnoticeable flicker covert CSC system. Wengrowski *et al.* [105] modified each colour pixel by shifting the base colour to a specific colour gradient which was then used to map pixels in an arbitrary image. Sato *et al.* [106] proposed a blue colour difference modulation scheme and verified its feasibility experimentally. In [107], the modulated signals were added to the R, G and B pixels of the video content and demodulated by a high-resolution camera. Recently, Wang *et al*. [59] suggested a spatial visual modulation scheme and conducted the quality assessment among 21 people to test the impact of modulated images to human perception. A recent vectorized colour modulation work extended the traditional colour modulation to a colour vector space by exploring all possible feasible combinations of colour vector pairs to represent more information [63]. The set to achieve the best demodulation performance under colour cross-talk was found through extensive search. Table 3 summarizes the key characteristics of these covert CSC systems.

**Table 3.** Comparisons of different covert CSC systems.

year	data rates	distance (m)	BER	media	reference
2016	22 kbps	0.7 m	0.1	video	[32]
2019	11.52 kbits frame^−1^	0.9 m	8.6 × 10^−5^ − 8.5 × 10^−2^	image	[63]
2015	0.2–5 kbps	0.3–1.2 m	<0.1	image, video	[58]
2017	16.67 kbps	0.7–1.3 m	0.1	video	[59]
2015	150–360 kbps	0.6–5 m	0.1–0.2	video	[102]
2012	6.22 kbps	—	5.4 × 10^−2^	image	[103]
2015	9–11 kbps	15 cm	<0.1	video	[104]
2017	144 bits frame^−1^	0.5 m	0.3	image	[105]
2016	0.756 kbps	0.5 m	<0.1	video	[106]
2017	4.5 Mbps	—	2.8 × 10^−3^ − 0.3	video	[107]

Currently, these convert or embedded CSC systems make a trade-off between visual experience and communication performance including data rate and bit error rate (BER). However, with the booming demands of ultra-high-definition video or images, traditional embedding methods may not satisfy the requirement of visual quality. Therefore, more efficient embedding techniques need to be deeply investigated while keeping a better viewing experience.

## 5. Link blockage and non-line of sight solutions

**Figure 12.**
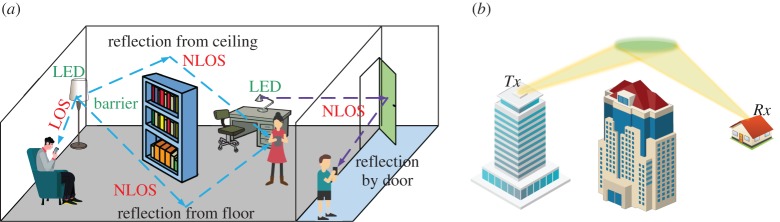
Typical NLOS OCC scenarios. (*a*) Indoor and (*b*) outdoor. (Online version in colour.)

Most reported OCC systems rely on a line of sight (LOS) path from the transmitter to the receiver. However, there are scenarios where the transmitter is not within the receiver’s FOV, for example a bookshelf blocks the transmission link between the lamp and smartphone in indoor, or two vehicles approach a cross-road in outdoor. In such cases, the connection cannot be established directly. Instead, an NLOS OCC system explores either indoor surface reflections or outdoor atmospheric scattering. Figure 12 depicts two typical NLOS OCC scenarios, where figure 12*a* is for an indoor environment and figure 12*b* is for an outdoor environment.

### (a) Indoor reflective non-line of sight

OCC is widely studied for indoor LOS communications. However, due to the limited FOV of a camera, an LOS-based OWC link typically suffers from shadowing and blockage, which limits the mobility and performance [109–111]. Thus, the OCC through an NLOS path emerges as a solution for achieving strong robustness to blocking and mobility. It mainly leverages the light reflections from walls, ceiling and other diffusely reflecting surfaces to establish the communication links, but may experience high path loss and multipath-induced inter symbol interference especially for links with high transmission rates.

For example, by detecting the rapid changes of LED radiation reflected from surfaces, Rajagopal *et al*. achieved indoor localization and an error-free communication purpose simultaneously at a data rate of 1.25 bytes s^−1^ and a lighting level above 600 lux [33]. In [112], an improved rolling shutter pattern demodulation algorithm was proposed based on reflected light from the wall. The system achieved an NLOS link distance of 1.5 m at a low illumination level of 145 lux in a dark environment. A novel 2 × *N* indoor NLOS OCC system was proposed and experimentally verified by Hassan *et al.* [113] with a specially designed packet structure and a detection methodology to support a 30 fps data transmission rate at 5 m. The impacts of ISO levels and exposure time on the transmitted power were investigated as well. Similarly, the NLOS space–time division multiplexing OCC system proposed by Hassan *et al.* performed well over a transmission distance of 10 m with a 12 mW transmitting power, and its tolerance to the ambient radiation was improved by mask matching and equal gain combining techniques [114].

Obviously, reflection-based NLOS OCC can maintain a stable communication link, or realize new forms of sensing and mobile computing with strong robustness to blocking and mobility. However, the path loss is usually much higher than that of an LOS link due to blockage, which limits the achievable data rate and transmission performance. Besides, the output of each pixel is a superposition of contributions from all light sources due to the NLOS nature, which includes additive interference [115].

### (b) Outdoor scattering non-line of sight

In outdoor scenarios, sometimes the transmitter beam is beyond the receiver FOV. For instance, vehicles moving in perpendicular directions in a crossroad are typically difficult to find each other. Tall buildings often shadow communication links. Thus, NLOS scattering communication is necessary. With the ubiquity of smartphones equipped with cameras and flash LEDs, OCC-based NLOS scattering communication emerges as a good candidate for outdoor key information delivery, such as steering information for vehicles, remote control instructions or an SOS signal for a trapped person in a barren field. Here, we will give a brief introduction to the feasibility of OCC-based NLOS scattering communication.

**Figure 13.**
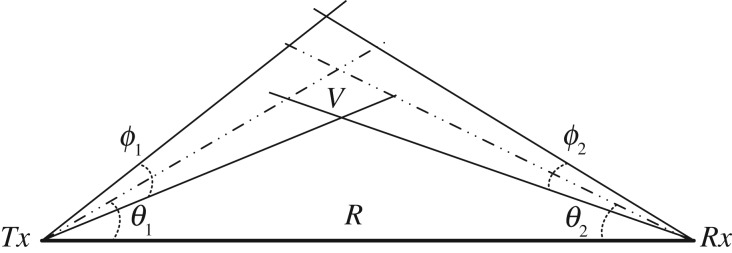
OCC-based NLOS scattering communication geometry.

Figure 13 shows a typical OCC-based NLOS scattering link geometry. The pitch angles of the transmitter and the receiver are denoted as *θ*_1_ and *θ*_2_, respectively. *ϕ*_1_ and *ϕ*_2_ are the beam width of the transmitter light source and the FOV of the receiver camera, respectively. *R* is the transmitter and receiver separation distance. *V* denotes the total interaction volume of the beam and FOV. Note that the azimuth of the transceiver is not considered here.

An NLOS channel model is usually analytically intractable, and the path loss *η*(*λ*) at a specific wavelength *λ* between the transmitter and the receiver can be obtained through a Monte Carlo method by simulating the propagation behaviour of each photon [116]. Incorporating the spectral responses of the LED *f*_LED_(*λ*) and camera *f*_Cam_(*λ*), respectively, in the VL spectrum from 380 to 780 nm, the average number of photons *λ*_ph_ arrived at the image sensor within the exposure time *τ* is given by

5.1 $$\lambda_{\text{ph}}=\frac{\tau P}{hc}\int_{\lambda }f_{\text{LED}}(\lambda )f_{\text{Cam}}(\lambda )\eta (\lambda )\lambda \;\text{d}\lambda ,$$

where *P* denotes the transmitted power corresponding to signal ‘1’ for OOK modulation, *h* is Planck’s constant and *c* is the speed of light. The received signal of an image sensor incorporating a signal-dependent noise model is given by Huang & Xu [117]

5.2 $$Y=X+XZ_{2}+\sqrt{X}Z_{1}+Z_{0},$$

where *X* is the desired signal component, and the second and third terms on the right are Gaussian noise terms, with mean zero and variances proportional to *X*^2^ and *X*, respectively, and the last term is a Gaussian noise term with zero mean and variance independent of *X*. In [116], an experiment in a dark room was conducted to verify the signal-dependent noise model in equation (5.2) for a practical grey sensor-based camera (Hamamatsu, C11440-52U). With 50 000 successive images taken at a fixed light intensity, Gaussian distribution with signal-dependent variance in a quadratic form of the signal was experimentally justified.

To further verify the feasibility of OCC-based NLOS scattering communication, an outdoor experiment was also conducted on the roof of two buildings, minimizing the influence induced by ambient noises [116]. At the transmitter, a pseudo random binary bit sequence was generated in Matlab, and transferred to the arbitrary waveform generator (AWG) (RIGOL DG5252). Then a white LED driven by a DC bias generated a 900 mW total optical power. The data rate was set to 100 bps, which was mainly limited by low sensor frame rate and the significant NLOS channel path loss. At the receiver, the frame rate of the camera was set to 800 Hz to achieve a better BER performance. Note that a higher frame rate causes decreased exposure time, reducing the number of received signal photons and background photons simultaneously. A lower frame rate decreases the achievable data rate but enhances the received photons. Thus, there is a tradeoff between the data rate and SNR. The pitch angle of the transceiver was set to 15° to facilitate coarse alignment and acquire a better performance. Then the BER was tested at different distance values (130 m and 300 m) at an eight times oversampling rate of the camera. From the measured pixel output values for captured ‘1’ and ‘0’ at different distance, their statistical distributions were found to fit the Gaussian distribution well. The variance of captured ‘1’ was found to be larger than that of ‘0’, and increase with the output mean value, indicating the nature of signal-dependent noise. It was also observed that the probability distribution curves corresponding to ‘1’ and ‘0’, respectively, were well separated, leading to an error-free reception during the observed long bit periods after downsampling and optimal detection from the captured signal.

To overcome the physical limitations from practical experiments, Monte Carlo simulations were also performed to predict range-dependent BERs for different system settings. A white LED with optical power 50 mW every 1 nm in the entire VL spectrum was assumed in the simulation. The NLOS scattering path loss over the entire LED spectrum for additional transceiver geometric parameter values was obtained by Monte Carlo simulations with the atmosphere parameters specified in [116]. At the receiver, the noise distribution parameters were derived from the experimental results. Then the BER performance was predicted at different transmission distance under various transceiver pitch angles and sensor exposure time.

**Figure 14.**
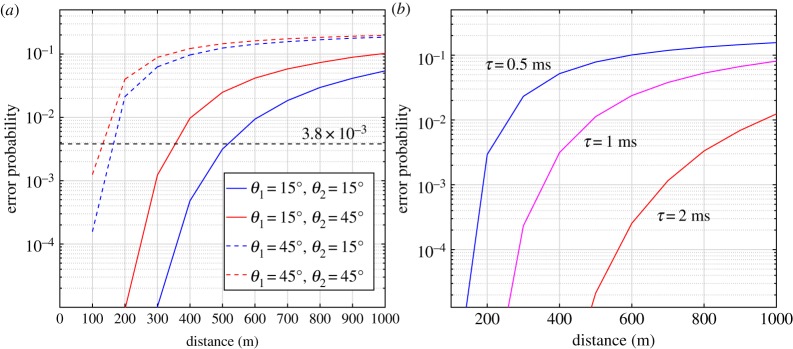
Predicted error probability for different (*a*) transceiver elevation angles and (*b*) exposure time. (Online version in colour.)

Figure 14 illustrates the predicted range-dependent BER for different system settings. Figure 14*a* provides BERs for distance ranging from 100 to 1000 m and various transceiver pitch angle pairs (*θ*_1_, *θ*_2_) = (15°, 15°), (15°, 45°), (45°, 15°), (45°, 45°) with a fixed exposure time *τ* = 1.25 ms. Clearly, the transmitter pitch angle has a greater impact on the system performance, which implies that longer distance can be achieved by lowering the transmitter pitch angle. The distance can be extended over 500 m considering the forward error correction (FEC) limit represented by a horizontal dashed line in figure 14*a*. Figure 14*b* depicts the range-dependent error probability for various exposure time values with a fixed pitch angle pair (*θ*_1_, *θ*_2_) = (15°, 15°). The exposure time here mainly determines the achievable maximum frame rate. Shorter exposure time results in a higher achievable frame refresh rate, but at a cost of reduced number of captured signal photons. It is clear that for a fixed optical SNR (keeping a fixed ratio of arrived signal photons and background noise photons), longer integral time within a symbol duration will yield better BER performance, but enhancement may be limited by the saturation of pixels in a practical imaging system.

## 6. Future directions

Despite numerous appealing features of the OCC technologies, there still remain several challenges for practical implementation. For example, existing works mainly focus on simplex communication while ignoring the full-duplex requirement for instant messaging or two-party interaction. But when full-duplex communication is considered, OCC may introduce interference which incurs more vulnerability to mobile transceivers. Besides, an OCC system may be operated under strong ambient radiation from different sources such as solar irradiance, street lamps or artificial advertising boards. Such interference degrades the system performance seriously, and even causes the image sensor to be saturated and blind. Furthermore, practical LEDs and screen pixels exhibit nonlinearity. The nonlinear distortion has to be compensated in system design, optimization and practical implementation in typical intensity modulation-based systems. And the blur issue exists [118] when a camera is not focused due to the absence of an adaptive focusing mechanism, leading to SNR degradation and poor spatial separation of source signals. Additionally, an ultra-high frame rate camera is still in an unaffordable price for general consumer electronics.

The above issues need to be further tackled from the devices to transmitter and receiver design, advanced signal processing, interference suppression, and system and network protocol designs, to fully enjoy the various benefits of the OCC technologies. To further improve the communication data rate, joint modulations integrating various domains, such as intensity, colour, spatial, phase, frequency, should be investigated in depth. It is necessary to further explore the possibility of developing higher performance image sensors to deliver a higher frame rate, higher resolution, wider dynamic range and higher sensitivity.

As commonly acknowledged, the current RF spectrum can hardly fulfill the exponential growth in demand of wireless capacity for next-generation communications. The optical spectrum will play an important role for communications in offices, shopping malls, industrial areas, public gathering places, public transportation hubs and many others. OCC appears as one of the most attractive options in terms of flexibility, mobility, popularity and cost.

## Data accessibility

This article has no additional data.
